# *Candida albicans* quorum-sensing molecule farnesol modulates staphyloxanthin production and activates the thiol-based oxidative-stress response in *Staphylococcus aureus*

**DOI:** 10.1080/21505594.2019.1635418

**Published:** 2019-07-06

**Authors:** Taissa Vila, Eric F. Kong, Ahmed Ibrahim, Kurt Piepenbrink, Amol C. Shetty, Carrie McCracken, Vincent Bruno, Mary Ann Jabra-Rizk

**Affiliations:** a Department of Oncology and Diagnostic Sciences, Dental School, University of Maryland, Baltimore, MD, USA; b Department of Microbiology and Immunology, School of Medicine, University of Maryland, Baltimore, MD, USA; c Department of Pharmaceutical Sciences, School of Pharmacy, University of Maryland, Baltimore, MD, USA; d Department of Food Science and Technology and Nebraska Food for Health Center, University of Nebraska, Lincoln, NE, USA; e Department of Biochemistry, University of Nebraska, Lincoln, NE, USA; f Center for Integrated Biomolecular Communication, University of Nebraska, Lincoln, NE, USA; g Institute for Genome Sciences, University of Maryland School of Medicine, Baltimore, MD, USA

**Keywords:** Fungal-bacterial interactions, quorum sensing, farnesol, transcriptional modulations, pathogenesis, biofilm

## Abstract

Microbial species utilize secreted-signaling molecules to coordinate their behavior. Our previous investigations demonstrated a key role for the *Candida albicans*-secreted quorum-sensing molecule farnesol in modulating *Staphylococcus aureus* response to antimicrobials in mixed biofilms. In this study, we aimed to provide mechanistic insights into the impact of farnesol on *S. aureus* within the context of inter-species interactions. To mimic biofilm dynamics, farnesol-sensitized *S. aureus* cells were generated *via* sequential farnesol exposure. The sensitized phenotype exhibited dramatic loss of the typical pigment, which we identified as staphyloxanthin, an important virulence factor synthesized by the Crt operon in *S. aureus*. Additionally, farnesol exposure exerted oxidative-stress as indicated by transcriptional analysis demonstrating alterations in redox-sensors and major virulence regulators. Paradoxically, the activated stress-response conferred *S. aureus* with enhanced tolerance to H_2_O_2_ and phagocytic killing. Since expression of enzymes in the staphyloxanthin biosynthesis pathway was not impacted by farnesol, we generated a theoretical-binding model which indicated that farnesol may block staphyloxanthin biosynthesis *via* competitive-binding to the CrtM enzyme crucial for staphyloxanthin synthesis, due to high structural similarity to the CrtM substrate. Finally, mixed growth with *C. albicans* was found to similarly induce *S. aureus* depigmentation, but not during growth with a farnesol-deficient *C. albicans* strain. Collectively, the findings demonstrate that a fungal molecule acts as a redox-cycler eliciting a bacterial stress response *via* activation of the thiol-based redox system under the control of global regulators. Therefore, farnesol-induced transcriptional modulations of key regulatory networks in *S. aureus* may modulate the pathogenesis of *C. albicans-S. aureus* co-infections.

## Importance

The fungus *Candida albicans* and the bacterial species *Staphylococcus aureus* are important microbial pathogens often co-isolated from hospitalized patients. We have previously demonstrated that these species actively interact, as they co-exist in a mixed biofilm. In this study, we aimed to provide mechanistic insights into the role of a secreted fungal molecule in mediating the interactions between these diverse species, and the impact of these interactions on the behavior of the bacterial cell. The findings demonstrated that the molecule secreted by *C. albicans* inhibits the synthesis of a pigment considered an important virulence factor in *S. aureus*. Importantly, transcriptional analysis of the bacterial cells demonstrated that the secreted molecule also modulates the expression of virulence-associated genes in *S. aureus*. Understanding the mechanisms of the interactions between these diverse pathogens is crucial in terms of pathogenesis and drug resistance of biofilm-associated polymicrobial infections.

## Introduction

Microbial species utilize secreted-signaling chemical molecules to coordinate their collective behavior, particularly within polymicrobial biofilms. The opportunistic fungal and bacterial pathogens *Candida albicans* and *Staphylococcus aureus*, respectively, are the most frequent combination of organisms co-isolated from various niches in the human host [1–6]. Our previous *in vitro* and *in vivo* studies have characterized dynamic and complex interactions between these species with important clinical and therapeutic implications [7–11]. More recently, we demonstrated that in a mixed biofilm, the matrix composed of secreted fungal cell wall polysaccharides, conferred co-existing *S. aureus* cells with enhanced tolerance to antimicrobials [12]. However, findings also indicated that other *C. albicans* secreted effectors also play a central role in mediating the process, which we identified to be the secreted quorum-sensing molecule farnesol [11].

In biofilms, quorum sensing (QS) or cell-cell communication is a crucial process mediated by secreted molecules, which allows one species to detect and respond to the presence of another, favoring concerted behavior in response to changing conditions. Therefore, these secreted mediators can affect cell physiology and may assume vital importance particularly within a polymicrobial environment, which facilitates cross-exposure to these molecules [13,14]. One of the best-characterized QS molecules is the tetraprenoid farnesol, a key derivative in the sterol biosynthesis pathway in eukaryotic cells. In *C. albicans*, farnesol is endogenously generated by enzymatic dephosphorylation of farnesyl diphosphate (FPP) and secreted into the environment [11,15–17]. Although farnesol was shown to play a central role in *C. albicans* physiology, we previously demonstrated that exposure to exogenous farnesol at above threshold levels triggers a process of apoptosis in eukaryotic cells, which was preceded by the accumulation of intracellular reactive oxygen species (ROS) [18,19].

Bacteria have evolved an effective oxidative-stress response that includes the production of ROS-detoxifying enzymes such as catalases, alkyl hydroperoxidases, and superoxide dismutases [20]. Another line of defense against oxidative stress includes the production of antioxidant carotenoid pigments that can quench ROS. In *S. aureus*, the carotenoid pigment staphyloxanthin (STXN) is considered an important virulence factor due to its antioxidant activity by contributing to survivability against the oxidative burst derived from immune cells [21,22]. Therefore, although not vital for *S. aureus* growth, this pigment has been implicated in fitness against oxidative stress [20]. To overcome the deleterious effects of oxidative stress, staphylococci employ a multitude of defense strategies controlled by a complex network of global regulators. Therefore, based on our combined previous investigations including the most recent demonstrating that farnesol modulates *S. aureus* cells response to antimicrobials [11], in this study, we aimed to elucidate the global impact of farnesol on *S. aureus* on a transcriptional level. Additionally, the close structural homology of farnesol to farnesyl diphosphate (FPP), the substrate for the CrtM enzyme responsible for STXN biosynthesis [23], led us to hypothesize that farnesol could disrupt STXN production *via* competitive binding to CrtM.

Given the demonstrated synergistic interactions between *C. albicans* and *S. aureus* in a mixed biofilm where farnesol is continuously secreted by *C. albicans*, it is highly likely that exposure to farnesol impacts the bacterial cell physiology and behavior. However, although farnesol’s role in orchestrating *C. albicans* growth in biofilm is well studied, its effect on *S. aureus* within the context of inter-species interactions has not been explored. With *C. albicans* and *S. aureus* receiving renewed attention because of the escalating development of polymicrobial infections and increase in the emergence of strains resistant to antimicrobials, it has become crucial to understand the mechanisms and the clinical relevance of their interactions [3,24,25]. To that end, in this study, we aimed to expand on our previous investigations to further elucidate the impact of this fungal secreted molecule on *S. aureus* physiology.

## Results

### Farnesol dose-dependent effect on cell pigmentation

**Figure 1.**
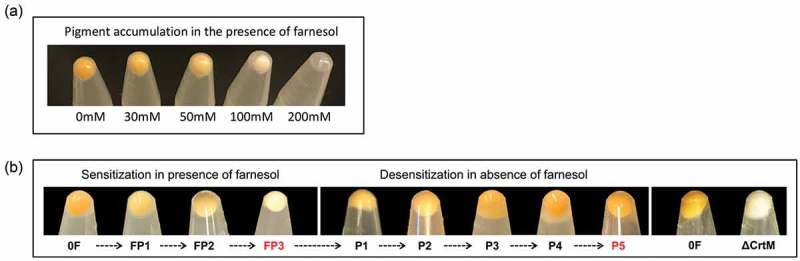
(a) Dose-dependent depigmentation of *S. aureus. S. aureus* cells grown in the presence of different concentrations of farnesol showed dose-dependent loss of pigmentation. (b) Farnesol sensitization and desensitization of *S. aureus* modulate cell pigmentation. Sensitization of *S. aureus* cells through daily passaging (P) in media supplemented with 50 µM of farnesol resulted in progressive loss of pigment with full depigmentation occurring at the third passage (FP3). However, desensitization of cells through passaging in farnesol-free media restored pigmentation (P5). The farnesol-induced depigmentation in the farnesol-sensitized *S. aureus* cells (FP3) is comparable to that in the *S. aureus* mutant strain (∆crtM) lacking staphyloxanthin. 0F are control cells not exposed to farnesol.

*S. aureus* cells grown in the presence of different concentrations of farnesol exhibited dose-dependent loss of pigmentation with a total loss of pigment accumulation observed when cells were exposed to 100 µM of farnesol for 16 h. As expected, concentrations higher than 100 µM were toxic and impaired growth (Figure 1(a)).

### Generation of a depigmented *S. aureus* phenotype upon repeated exposure to farnesol (sensitization)

In order to more closely reflect the dynamics in a mixed biofilm where *S. aureus* cells are continually exposed to farnesol secreted by *C. albicans* over time, *S. aureus* cells were passaged in culture media supplemented with farnesol to generate “sensitized” cells. It was observed that the farnesol-sensitized cells recovered during the passaging process exhibited progressive loss of the typical yellow color with complete depigmentation seen upon third passaging in farnesol (FP3) (Figure 1(b)).

### Reversion to pigmented phenotype upon gradual removal of the sensitized cells from farnesol (desensitization)

To determine whether the observed farnesol-mediated depigmentation event is a transient-conditional state, the farnesol pressure was gradually removed by passaging the depigmented sensitized cells (FP3) through farnesol-free culture media to generate a “desensitized” phenotype (P5). Comparison of cell pellet color at each passage demonstrated gradual restoration of pigment to level comparable to that in control cells (not exposed to farnesol) upon fifth passaging (Figure 1(b)).

### Farnesol-induced depigmentation in sensitized cells is comparable to that in the staphyloxanthin-deficient *S. aureus ∆crtM* mutant strain

Staphyloxanthin (STXN) is the most prominent pigment in *S. aureus* responsible for the typical yellow color and the *S. aureus ∆crtM* mutant strain lacking the CrtM enzyme responsible for STXN synthesis is depigmented. Using this mutant as a control for pigment comparison, the observed dramatic loss of color in the sensitized cells was found to be comparable to that of the STXN lacking strain (Figure 1(b)).

### Identification of the pigment as STXN by HPLC analysis

**Figure 2.**
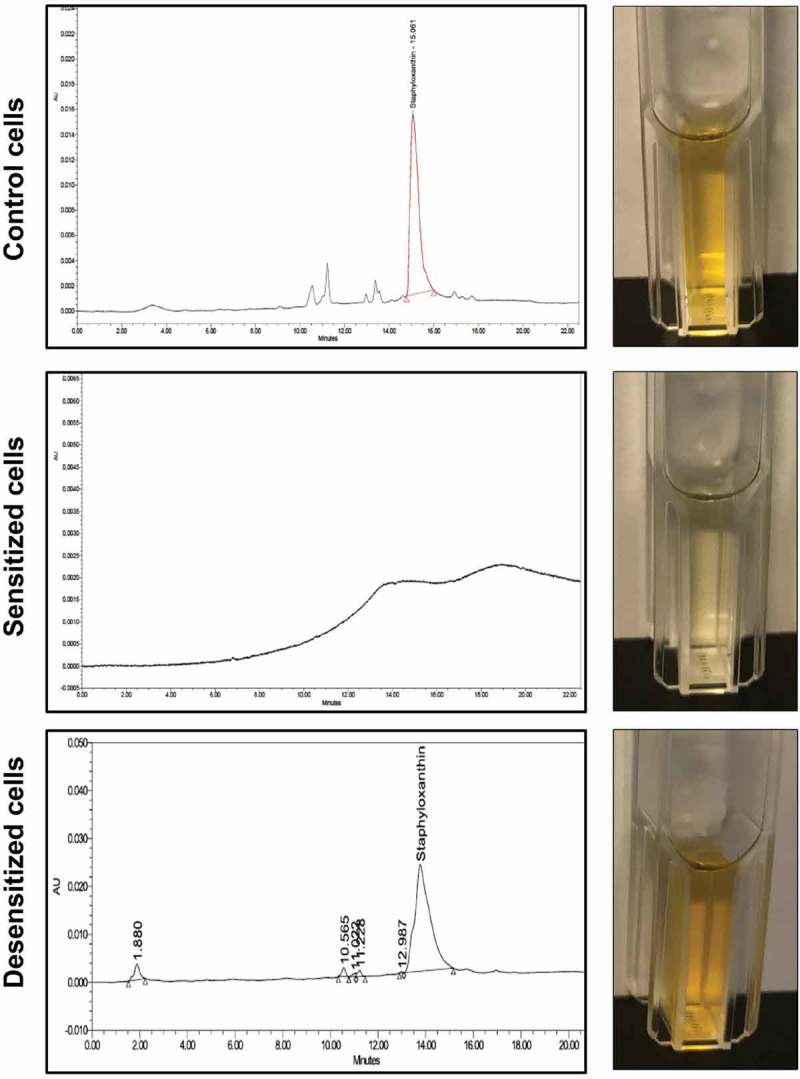
HPLC analysis of cell extracts from sensitized and desensitized cells identifies the pigment as staphyloxanthin (STXN). Ethanol extracts from control, farnesol-sensitized, and desensitized cells were monitored at 450 nm. Based on the resultant chromatograms, a peak with an average retention time of 15.033 min, consistent with that reported for STXN, was detected in extracts from control cells but not from farnesol-sensitized cells. In contrast, STXN peaks similar to those in control cells were detected in the farnesol-desensitized cells, indicating restoration of the pigment upon removal of farnesol supplementation from the media. HPLC analyses were obtained from three independent, biological replicates; representative chromatograms and images of extracts are shown.

To identify the *S. aureus* cell pigment impacted by farnesol, HPLC analysis was performed on cell extracts from sensitized and desensitized cells, alongside their respective controls. Based on the resultant chromatograms, all control extract samples displayed a significant signal at 450 nm with an average retention time of 15.033 min, consistent with that reported for STXN [23]. In contrast, no peaks were detected in the extracts from the farnesol-sensitized cells. However, extracts from the desensitized cells displayed the same STXN peak seen in control samples (Figure 2).

### Farnesol-induced ROS and its effect on pigmentation

**Figure 3.**
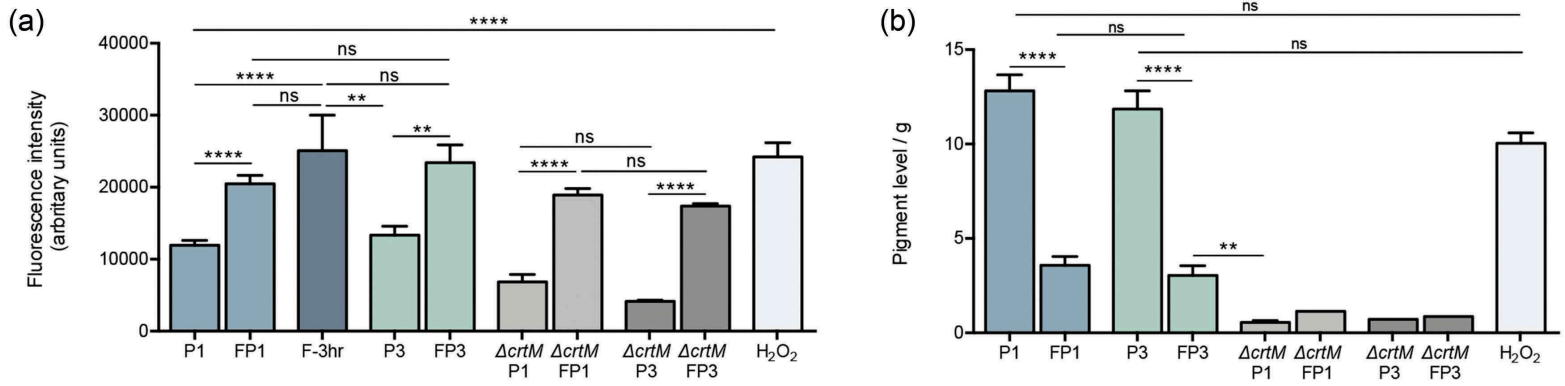
Intracellular ROS accumulation and depigmentation of *S. aureus* cells grown under various conditions. (a) ROS accumulation was quantitatively assessed based on measurement of the intensity of fluorescence following staining with CellROX^TM^ green, which becomes fluorescent after selective reduction by intracellular ROS. Cells were grown for 18 hrs in the absence (P1) or presence of farnesol (FP1) or H_2_O_2_; similarly, ROS was comparatively assessed in the sensitized cells (FP3) and their control cells (P3); to assess the rapid response of cells to farnesol exposure, cells were also grown with farnesol for 3 hr and ROS accumulation was measured. The *∆crtM* mutant was also serially exposed to farnesol and ROS accumulation was evaluated following first (*∆crtM* FP1) and third passage (*∆crtM* FP3) with *∆crtM* P1 and *∆crtM* P3 serving as respective controls. Results demonstrated rapid induction of ROS within 3 hr of exposure to farnesol, which although remained significantly higher than in control cells, was lower in the cells exposed to farnesol overtime (FP1 and FP3) compared to their respective controls (P1 and P3). Similarly, farnesol also induced ROS production in the *∆crtM* mutant. ROS level in the H_2_O_2_ exposed cells was comparable to that induced by farnesol. (b) Simultaneous measurement of pigment from extracts of cells demonstrated a significant decrease in pigment production in the farnesol-exposed cells (FP1, FP3). Growth with H_2_O_2_ for 18 hrs had no effect on pigment production compared to controls (P1 and P3) while, as expected, the *∆crtM* mutant showed no pigmentation. * *p* < 0.05; ** *p* < 0.01; *** *p* < 0.001; **** *p* < 0.0001; (ns): not significant.

To explore whether farnesol acts as an oxidative stressor, the level of ROS accumulation in cells under various conditions was measured. Results demonstrated significant spike in intracellular ROS within 3 hr of exposure to farnesol, which although decreased in the cells from first (FP1) and third passaging (FP3; sensitized cells), remained significantly higher compared to respective controls (P1 and P3) (Figure 3(a)). Cells grown with sub-lethal concentrations of H_2_O_2_ similarly exhibited ROS accumulation at a level comparable to that induced by farnesol (Figure 3(a)). Interestingly, although the *∆crtM* mutant also accumulated intracellular ROS when exposed to farnesol (*∆crtM* FP1 and *∆crtM* FP3), the level of accumulation did not increase with passaging (Figure 3(a)). Since STXN is an ROS quencher, simultaneous measurement of pigment from extracts of cells demonstrated a significant decrease in pigment upon initial exposure (FP1) and in the sensitized cells (FP3) (Figure 3(b)). In contrast, growth in the presence of H_2_O_2_ although induced ROS production, no effect on pigmentation (*p* > 0.05 compared to both P1 and P3) was seen, indicating that the decrease in pigment is primarily mediated by farnesol (Figure 3(b)). As the pigment is a secondary metabolite, growth in the presence of farnesol for 3 hr was not long enough to impact pigment production. As expected, the *∆crtM* mutant showed no pigment accumulation, which was not affected by passaging with farnesol (Figure 3(b))

### Farnesol-sensitized cells do not exhibit defects in growth rate, adhesion, or biofilm formation

**Figure 4.**
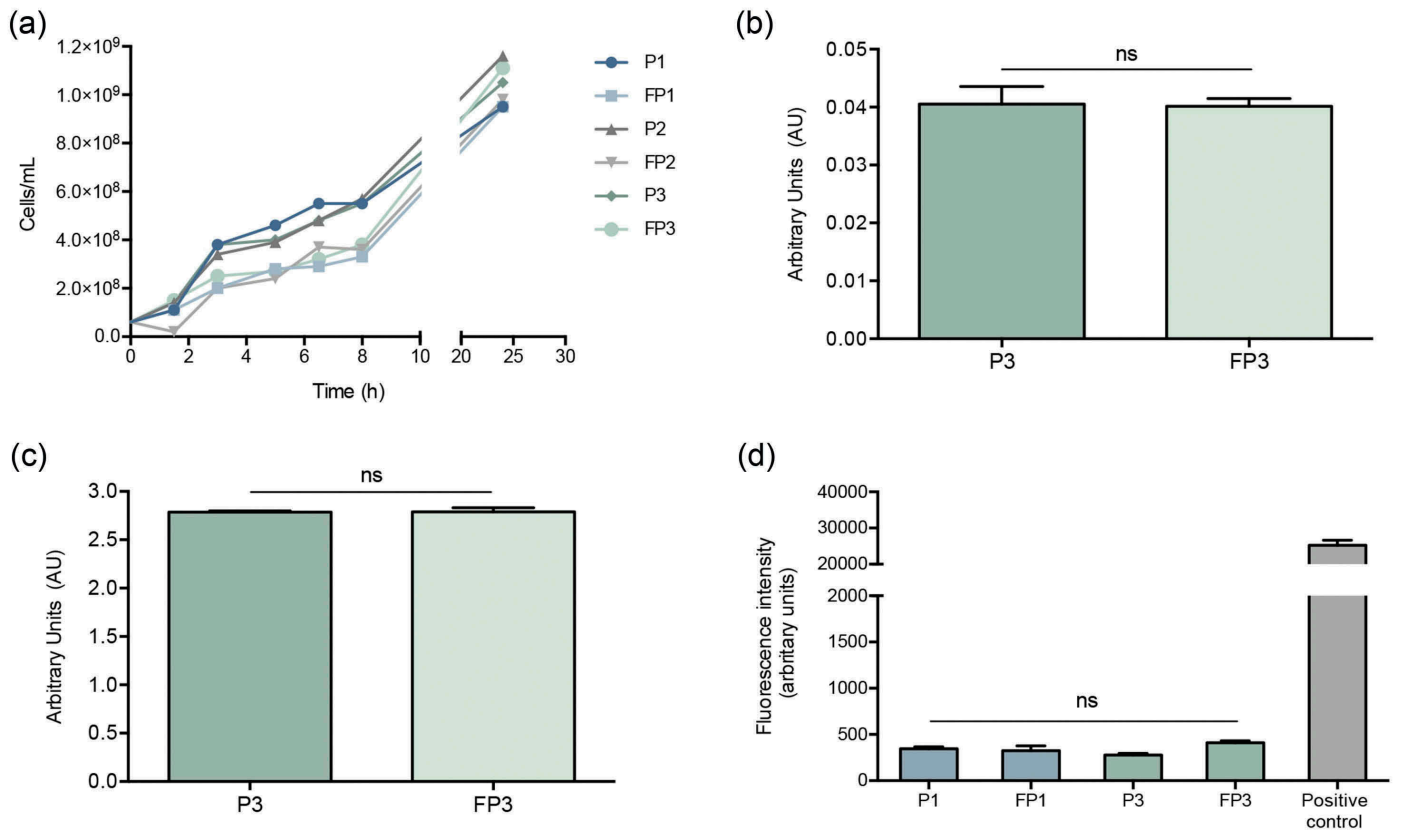
Farnesol-sensitized cells (FP3) do not exhibit defects in growth rate, adhesion or biofilm formation compared to their control cells (P3). (a) In order to determine if farnesol sensitization exerts an adverse effect on the cells, the growth rate was evaluated during the sensitization of cells. Based on optical density measurements, the farnesol-sensitized cells (FP1, FP2, and FP3) displayed similar logarithmic growth curves with a modest lag in growth observed within the first 8 hrs which subsequently, was comparable to that of respective control cells (P1, P2, and P3) indicating no apparent growth defects. The MTS metabolic assay was used to comparatively assess adherence and biofilm formation capabilities following a 90-min adhesion period and 24 hr biofilm growth. Results indicated no apparent defects in the sensitized cells (FP3) in (b) adhesion or (c) biofilm formation compared to control cells (P3). (ns): not significant. (d) Leakage assay indicates no disruption in cell membrane integrity in the farnesol-sensitized cells. Quantitative fluorimetric assay showed no significant differences between the farnesol treated (FP1, FP3) and control (P1, P3) cells in the level of internalized propidium iodide indicating no damage to the cell membrane. Cells exposed to 70% ethanol were used as positive controls.

In order to determine if farnesol sensitization exerts an adverse effect on the cells, the growth rate of cells was evaluated throughout the farnesol sensitization process. Based on optical density measurements, in the presence of farnesol, although a modest lag in growth was seen during the first 8 hrs, over the subsequent 24-hr growth rate was comparable to that of control cells indicating no growth defects (Figure 4(a)). Further, adherence and biofilm formation capabilities of sensitized cells were assessed following a 90-min adhesion period and 24 hr biofilm growth by the MTS metabolic Tetrazolium salt assay (Figure 4(b, c)) and CFU counts (data not shown). Results indicated no apparent defects in adhesion or biofilm formation in the sensitized cells.

### Farnesol-sensitization does not compromise cell integrity or induce morphological changes in the cells

**Figure 5.**
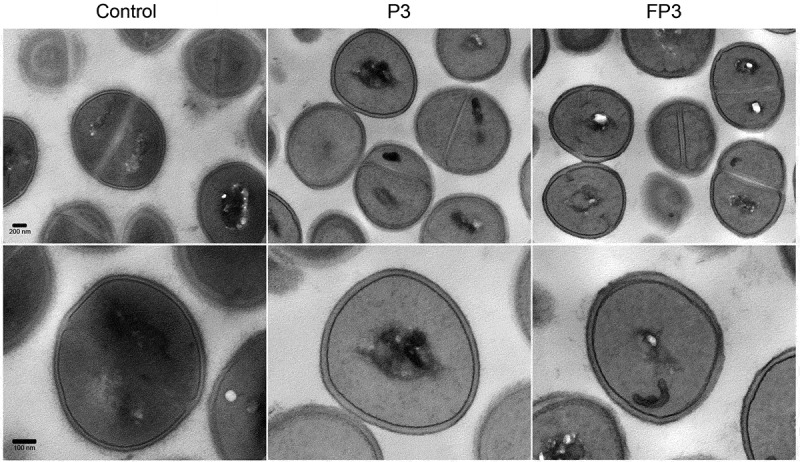
Transmission electron micrographs of farnesol-sensitized cells demonstrating no adverse effects on cell morphology. Farnesol-sensitized cells (FP3) were processed for TEM analysis in order to determine if farnesol-sensitization compromises cell integrity. Images revealed no noticeable abnormalities in the cell wall structure and overall cell morphology in sensitized cells (FP3) when compared to their passaged control cells (P3) and cells from 18-hr culture (control).

Since STXN accumulates in the cell wall, to affirm that the observed loss of pigment is not due to farnesol-induced disruption in cell membrane integrity, the sensitized cells were evaluated using propidium iodide quantitative fluorescent leakage assay. Results demonstrated no significant differences between the farnesol treated (FP1, FP3) and control (P1, P3) cells in the level of internalized propidium iodide indicating no damage to the cell membrane. Cells exposed to 70% ethanol were used as positive controls (Figure 4(d)). Additionally, sensitized cells were also subjected to transmission electron microscopy (TEM) analysis. Compared to control cells, images revealed no observable morphological or cell wall abnormalities in the sensitized cells (Figure 5).

### Farnesol induces major transcriptional modulations in *S. aureus* indicative of activation of the thiol-based redox system

To gain some mechanistic insight into the observed effects of farnesol on the bacterial cell, a comparative transcriptional analysis was performed on the sensitized cells. Specifically, we performed RNA-seq analysis on farnesol-sensitized cells (FP3) and their time-matched negative controls. Results of analysis identified a total of 769 genes with differential expression (fold change ≥2; *p* < 0.05) between the farnesol-sensitized and control samples. In this study, we were particularly interested in those genes that are known to be associated with the response to oxidative stress, listed in Table 1. Most notable among the up-regulated genes in the sensitized cells were redox sensors and global regulators involved in ROS detoxification *via* the thiol-specific redox system. Specifically, oxidant sensors and redox regulators (SarA, Fur/PerR family) and the thiol-based redox sensor Spx that induce transcription of antioxidant enzymes such as superoxide dismutases (SODs), catalase (KatA) and alkyl hydroperoxide reductase (Ahp), along with the enzymes under their control, were significantly upregulated. Although involved in redox sensing and response to oxidative stress, these global regulators also confer resistance to antibiotics and are important for the regulation of virulence. In contrast, genes involved in iron homeostasis, including surface determinants (Isd) involved in iron acquisition and transport, scavenger of intracellular iron (Dps), and proteins involved in iron binding (ferritin) and transport were down-regulated whereas the transcriptional regulator and ferric uptake repressor Fur was upregulated indicating attenuation of the iron uptake system. In addition to iron, CopA, which maintains copper homeostasis was also upregulated. Further, expression of enzymes involved in cell damage repair such as protein repair (thioredoxin/thioredoxin reductase; methionine sulfoxide reductases), DNA protection and repair (RecA, RecF, dps), and iron-sulfur (Fe-S) clusters repair mechanisms (*scdA)* were significantly increased in the sensitized cells. Not surprisingly, stress and heat shock proteins were among the genes significantly upregulated. Importantly, the transcriptional levels of CrtM and CrtN, the two key enzymes in STXN biosynthesis pathway, were not modulated by farnesol (Table 1).

**Table 1.** Functional classification of *S. aureus* genes differentially regulated in farnesol-sensitized cells compared to control cells. RNA-sequencing was used to assess gene expression in the sensitized cells.

Gene Symbol	LFC (log fold change)	p-value	ORF description
**Detoxifying enzymes and protection against oxidative stress**			
sodA	1.651	2.12E-06	Superoxide dismutase
ahpC1	2.812	2.85E-13	Alkyl hydroperoxide reductase subunit C
katA	2.136	2.16E-09	Catalase
bcp	0.682	0.106	Peroxiredoxin
ahpF	2.446	3.07E-10	Alkyl hydroperoxide reductase subunit F
acnA	1.971	4.62E-08	Aconitate hydratase
acsA1	1.498	7.76E-05	Acetyl-CoA synthetase
cysK	2.419	5.32E-11	Cysteine synthase
**Stress and heat shock proteins**			
asp23	3.256	2.14E-18	Alkaline shock protein 23
dnaJ	1.963	5.37E-08	Chaperone protein DnaJ
dnaK	2.489	3.43E-08	Molecular chaperone DnaK
grpE	3.216	1.17E-18	Heat shock protein GrpE
hrcA	3.526	5.30E-20	Heat shock transcriptional repressor
USA300HOU_0544	3.154	1.00E-15	Chaperone protein HchA
USA300HOU_1692	3.134	3.38E-15	Universal stress protein
USA300HOU_1697	2.657	5.16E-13	Universal stress protein
**Metal homeostatis**			
copA	2.350	8.48E-11	P-ATPase superfamily P-type ATPase copper transporter
htrA	−1.49389	0.00473613	Hemin ABC transporter membrane protein
htrB	−1.28848	0.026327736	Hemin ABC transporter ATP-binding protein
isdA	−1.449	0.01306	Iron-regulated surface determinant protein IsdA
isdC	−2.311	0.00605	Iron-regulated surface determinant protein IsdC
isdE	−1.640	0.00629	Iron ABC transporter binding protein
isdF	−1.760	0.00720	Iron ABC transporter membrane protein
isdG2	−1.814	0.00882	Heme-degrading monooxygenase IsdG
sirB	−1.649	0.00885	Iron ABC transporter membrane protein
USA300HOU_0232	−1.597	0.01001	Iron ABC transporter membrane binding protein
USA300HOU_1891	4.386	1.65E-29	Ferritin
USA300HOU_2544	−1.188	0.0072	FeoB family ferrous iron uptake protein
USA300HOU_0759	−1.532	0.0056	Iron ABC transporter membrane protein
**DNA protection and repair**			
recA	1.082	0.0013	Recombinase A
recF	1.114	0.0012	Recombination protein F
dps	2.625	6.99E-13	Dps family stress protein
**Protein damage repair**			
scdA	1.087	0.0191	Cell wall biosynthesis protein ScdA
msrA1	0.716	0.0716	Methionine sulfoxide reductase A
msrB	1.208	0.0133	Methionine sulfoxide reductase B
trxA	1.395	7.41E-05	Thioredoxin
trxB1	0.669	0.0782	Thioredoxin-disulfide reductase
trxB2	−0.563	0.2618	Thioredoxin-disulfide reductase
groEL	1.833	3.81E-07	Chaperonin GroEL
**Sensors and transcriptional regulators**			
sarA	3.380	1.22E-12	Accessory regulator A
USA300HOU_1499 (fur)	1.790	0.00014	Fur family transcriptional regulator
spxA	1.363	6.02E-05	Transcriptional regulator Spx
USA300HOU_2510	−1.930	3.22E-06	MarR family transcriptional regulator
USA300HOU_0709	1.247	0.00045	MarR family transcriptional regulator
USA300HOU_2502	−2.789	1.92E-05	MarR family transcriptional regulator
USA300HOU_1186	1.266	0.00104	Transcriptional repressor CodY
saeR	1.234	0.00267	Response regulator SaeR
rot	2.397	8.49E-08	Repressor of toxins Rot
agrA	1.244	0.012	Accessory gene regulator protein A
**Staphyloxanthin biosynthesis**			
crtM	0.162	0.851	Squalene desaturase
crtN	−0.066	0.848	Squalene synthase

### Farnesol-sensitized cells are less susceptible to killing by H_2_O_2_

**Figure 6.**
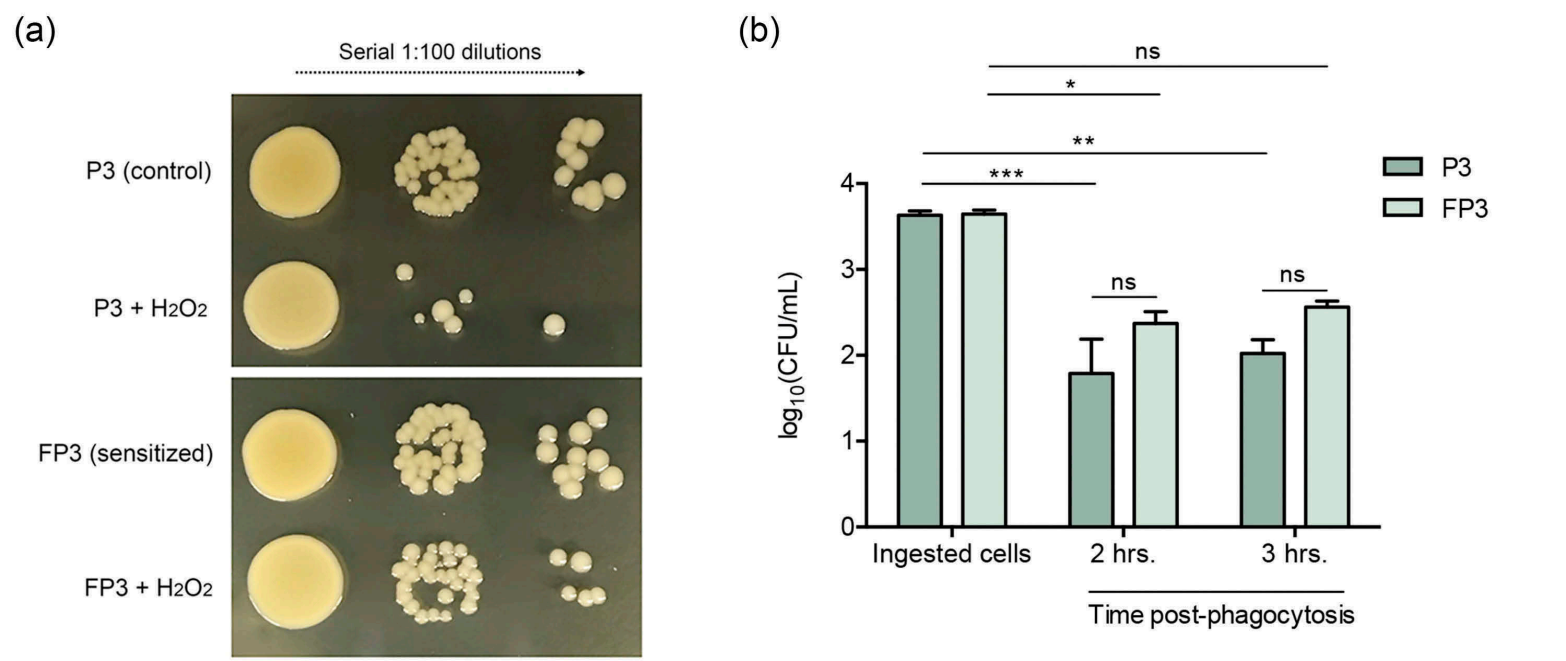
(a) Farnesol-sensitized cells exhibit decreased susceptibility to killing by H_2_O_2_. Killing assays demonstrated decreased susceptibility of the sensitized cells (FP3) to H_2_O_2_ compared to their control cells (P3). (b) Farnesol-sensitized cells show reduced susceptibility to phagocytic killing. *In vitro* macrophage phagocytosis assays demonstrated an increase in survival of sensitized cells (FP3) within phagocytic cells compared to control cells (P3). FP3 also showed reduced susceptibility to macrophage killing at 3 hrs post-phagocytosis compared to the total ingested cells (0 h). * *p* < 0.05; ** *p* < 0.01; *** *p* < 0.001; (ns): not significant.

To evaluate the impact of the transcriptional activation of the oxidative-stress response machinery on the cell response to oxidative stress, the susceptibility of the sensitized cells to the oxidizing agent H_2_O_2_ was evaluated. Based on results from killing assays, the sensitized cells (FP3) exhibited decreased susceptibility to H_2_O_2_ killing compared to their control cells (P3) (Figure 6(a)).

### Farnesol-sensitized cells are less susceptible to macrophage phagocytic killing

To explore the implications of the observed decrease in the susceptibility of the farnesol-sensitized cells to H_2_O_2_ killing within the context of host immune response, we performed *in vitro* macrophage phagocytosis assays. Evaluation of survival of phagocytosed *S. aureus* cells over the course of 3 hrs based on CFU counts demonstrated 65–70% post-phagocytosis survival for farnesol-sensitized cells (FP3) and 49–56% for control cells (P3) relevant to the number of ingested cells (100%). Compared to P3 cells, FP3 cells exhibited approximately 30% increase in survival (Figure 6(b)).

### Computational modeling predicts farnesol-CrtM binding

**Figure 7.**
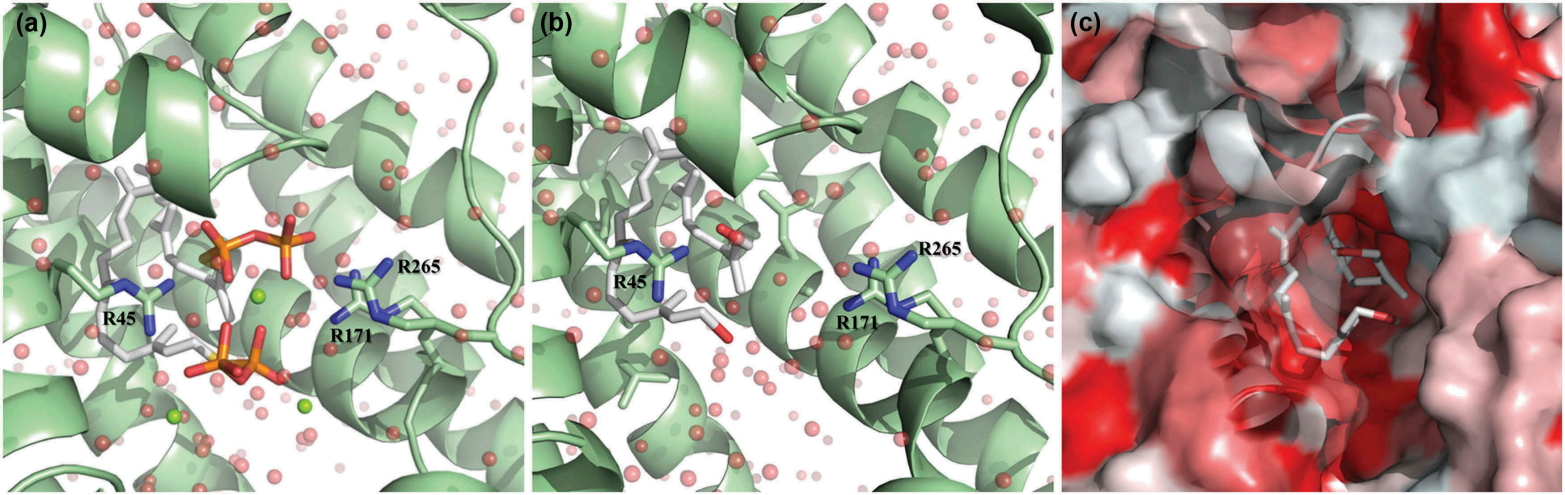
Theoretical model of farnesol binding to the CrtM enzyme responsible for staphyloxanthin synthesis. The high-resolution crystal structure of CrtM bound to its substrate farnesyl diphosphate was utilized to model how farnesol may interact with CrtM. (a) CrtM (green) bound to farnesyl thiopyrophosphate (from PDB ID: 3W7F); magnesium counter-ions are depicted as green spheres; the modeled solvation sphere is shown as semitransparent red spheres. (b) Model of CrtM bound to farnesol. The terminal sulfur-diphosphate of the farnesol backbone was replaced with a hydroxyl and the interface was resolved. (c) Hydrophobicity plot of the CtrM-farnesol model. The surface of CtrM surface is depicted in a hydrophobicity scale ranging from true red (most hydrophobic) to white (most hydrophilic). Unlike the altered electrostatics shown in panels A and B, the farnesol backbone is predicted to interact identically with the large hydrophobic (red) patches of CrtM in the interior of the pseudo-dimeric interface.

Due to the close structural similarity of farnesol to FPP, the substrate for the *S. aureus* CrtM enzyme, we modeled the structure of the hypothetical CtrM/farnesol complex using the high-resolution crystal structure of CrtM bound to the inhibitor farnesyl thiopyrophosphate (FPS) (PDB ID: 3W7F) [26] (Figure 7(a)). After replacing the terminal sulfur-diphosphate with a hydroxyl, the interface was resolved [27] (Figure 7(b)). The majority of the interface was conserved in this model, consisting largely of aliphatic contacts in the interior of the pseudo-dimeric interface (Figure 7(c)). Unsurprisingly, the loss of the diphosphate results in the loss of multiple electrostatic contacts; of 49 hydrogen bonds (including solvent hydrogen bonds) found in the 3W7F crystal structure, 37 are unchanged in the CrtM/farnesyl model and 12 are absent. Six unique hydrogen bonds are formed in the CrtM/farnesyl model (Supplementary Table 1–4). Since the energetic gains from hydrogen bonds are offset by a desolvation penalty, solvent exclusion from large hydrophobic patches (~500 Å^2^ for the CrtM-farnesol interface) (Figure 7(c)) is commonly accepted to make a greater contribution more to the free energy of binding [28]. Correspondingly, we expect the affinity for CrtM for farnesol to be in the nanomolar range based on the inhibitor concentration of FPS for CrtM (~100 nM).

### Growth of *S. aureus* with *C. albicans* induces depigmentation but not growth with a farnesol-deficient *C. albicans* strain

**Figure 8.**
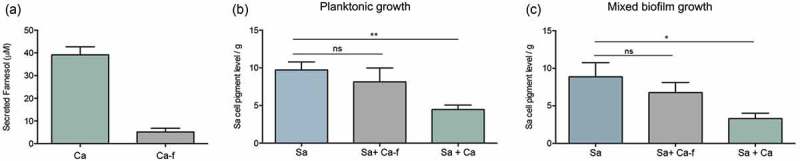
Impact of co-growth with *C. albicans* on *S. aureus* cell pigmentation. (a) HPLC measurement of farnesol secreted by *C. albicans* in biofilm spent culture media demonstrated ~75% reduction in farnesol in media from the deficient strain compared to farnesol-producing strain. (b) Pigment measurement from extracts of *S. aureus* cells grown planktonically with *C. albicans* demonstrated significant loss in pigment production during growth with *C. albicans* (Sa+Ca). In contrast, no significant changes in pigmentation in the *S. aureus* cells grown with the *C. albicans* farnesol-deficient strain (Sa+Ca-f). (c) Pigment measurement in extracts from *S. aureus* cells recovered from mixed biofilms with *C. albicans* similarly demonstrated significant loss in pigment production during growth with *C. albicans* (Sa+Ca) but not with the farnesol-deficient *C. albicans* strain (Sa+Ca-f). * *p* < 0.05; ** *p* < 0.01; (ns): not significant.

To begin to explore the physiological impact of *C. albicans*-secreted farnesol on *S. aureus* cells during mixed biofilm, dual-species biofilms were grown and recovered *S. aureus* cells were evaluated. In addition, in order to identify farnesol as the *C. albicans*-associated effector responsible for any observed modulations in *S. aureus* cell pigmentation, a farnesol-deficient *C. albicans* strain was also included in mixed growth experiments, performed under planktonic and biofilm growth conditions. Prior to these experiments, farnesol production by both *C. albicans* strains was evaluated *via* comparative measurement of farnesol secreted during *C. albicans* biofilm growth. Based on HPLC analysis of spent culture media of biofilms from both *C. albicans* strains, measured farnesol concentration averaged approximately 40µM in media from the farnesol-producing strain compared to less than 10µM in media from the farnesol-deficient strain indicating 75% reduction in farnesol production (Figure 8(a)). Planktonic co-culture experiments were performed where *in lieu* of exogenous farnesol supplementation, *S. aureus* was grown with the *C. albicans* strains. As expected, where *S. aureus* cells recovered from co-culture with the farnesol-producing *C. albicans* strain showed a dramatic loss of pigmentation, co-growth with the farnesol-deficient strain had no impact on *S. aureus* pigmentation (Figure 8(b)). In addition, mixed biofilms were also performed; strikingly, where *S. aureus* cells from single species biofilm exhibited the typical yellow cell color, those harvested from mixed biofilms with the farnesol-producing strain of *C. albicans* exhibited a significant loss in cell pigmentation. Conversely, cell pigmentation was retained when *S. aureus* was grown with a farnesol-deficient *C. albicans* strain (Figure 8(c)). *S. aureu*s cells demonstrated no significant differences in levels in ROS accumulation in the cells recovered from mixed biofilms compared to those recovered from biofilms of *S. aureus* (data not shown).

## Discussion

In a biofilm environment, microbial species are highly interactive and employ a range of complex cell-to-cell communications, particularly when they involve diverse organisms such as fungi and bacteria [14,29,30]. Based on our extensive investigations elucidating the interactions between *C. albicans* and *S. aureus*, our recent studies focused on investigating the role of farnesol in orchestrating inter-species communications. The findings demonstrated a phenomenon whereby exposure of *S. aureus* to farnesol conferred the bacteria with enhanced tolerance to antibacterials, which was partly attributed to upregulation of drug efflux pumps [12]. Combined with our previous studies, these recent findings led us to hypothesize that farnesol may act as a xenobiotic and oxidative stressor that not only modulates the response of the bacterial cell to antibacterials, but also impacts other aspects of bacterial cell behavior. To that end, in this study, we explored the effects of farnesol on *S. aureus* within the context of oxidative stress and activation of cell response machinery, with the overall goal of providing insights into this complex fungal–bacterial interaction. Therefore, to more closely mimic the dynamics within a mixed biofilm where farnesol is increasingly secreted by *C. albicans*, we generated a farnesol “sensitized” *S. aureus* phenotype through successive exposure to farnesol. Interestingly, it was observed that the sensitized cells exhibited loss of the typical cell yellow pigment, which we subsequently identified as staphyloxanthin, an important virulence factor in *S. aureus.*

In addition to the phenotypic effects on the cell, farnesol exposure also induced intracellular ROS accumulation. In bacteria, if the ROS burden is high and the general stress-response systems are overwhelmed, the SOS response under control of transcriptional regulators can become activated [20]. Paradoxically, an SOS response has been linked to antibiotic resistance, *via* upregulation of drug efflux pumps that function in the export of toxic substances [31,32]. However, the induced resistance could be transient in the presence of an effector, which was demonstrated in our previous study whereupon removal of farnesol pressure, *S. aureus* cells reverted to their original drug-susceptible phenotype [11]. Additionally, ROS accumulation can damage biological molecules such as DNA, amino acids, and proteins. Therefore, staphylococci have evolved a multitude of oxidative defense strategies controlled by a complex network of regulators, to detect and repair the damage caused by oxidative stress [20]. Specifically, exposure of bacteria to redox-active compounds disrupts cellular redox status resulting in thiol-disulfide switches. These switches in redox-sensing regulators activate detoxification pathways to restore the redox balance; however, these redox regulators are often also important virulence regulators [33,34]. Therefore, we hypothesized that farnesol is a redox cycler that exerts oxidative stress on the bacterial cell, activating an SOS response under control of transcriptional regulators. To that end, we performed transcriptome analysis of the farnesol-sensitized cells to elucidate the transcriptional responses to farnesol potentially impacting *S. aureus* fitness and pathogenesis, including those pertaining to STXN production.

As expected, RNA-sequencing analysis demonstrated major transcriptome alterations involving key regulatory pathways including redox sensors and global regulators involved in ROS detoxification *via* the thiol-specific redox system (Table 1). Most notable among the regulators modulated by farnesol are the Fur/PerR family, the MarR (multiple antibiotics resistance-type) redox regulators of the SarA subfamily, and the thiol-based redox sensors SpxA (listed in Table 2 with their regulon genes and functions) [20,34–37]. The PerR regulon includes many genes involved in the oxidative-stress response and iron storage, including *katA, ahpCF, mrgA, bcp*, and *trxA* genes, which were found to be upregulated. The activity of PerR is dependent upon metal ions and the ferric uptake regulator (Fur) is a transcriptional regulator that is partially responsible for maintenance of iron homeostasis during iron-limited growth [33,37]. Of significance, MarR redox regulators of the SarA subfamily confer resistance to antibiotics and ROS, and are important for the regulation of virulence. In fact, these regulators affect the transcription of as many as 350 genes including several multidrug efflux pumps and, therefore, function in resistance to antibiotics including vancomycin [20,38–42]. In our analysis, these regulators were found to be impacted by farnesol which is of particular significance in light of our recent findings demonstrating farnesol-mediated vancomycin resistance in *S. aureus* [11].

**Table 2.** Redox regulators in *S. aureus* and their regulon genes and functions.

The Fur/PerR-family of peroxide-specific redox regulators				
Redox sensor	Signal	Regulon genes	Regulon function	References
PerR	H_2_O_2_	ahpCF	Peroxiredoxin	Horsburgh et al. [37]
		katA	Catalase	
		mrgA	Miniferritin	
		fln	Ferritin	
		bcp	Bacterioferritin comigrating protein	
		trxA	Thioredoxin	
		fur	Fe-uptake repressor	
		perR	Peroxide repressor	
**The MarR-type redox regulators of the SarA subfamily**				
**Redox sensor**	**Signal**	**Regulon genes**	**Regulon function**	**References**
SarA	H_2_O_2_ Diamide	sodA	Superoxide dismutase	Ballal and Manna [44]
		trxB	Thioredoxin reductase	
		hla	α-hemolysin	
		spa	Protein A	
		fnb	Fibronectin-binding	
		can	Collagen-binding	Sun et al. [38]
		icaRA	Entertoxin C	
		ssp, aur	Biofilm formation	
		rot, agr, sarS, sarV, sarT	Virulence regulators	
**The Spx disulfide-stress-sensing regulators**				
**Redox Sensor**	**Signal**	**Regulon Genes**	**Regulon Function**	**References**
Spx	Diamide H_2_O_2_	trxAB	Thioredoxin/thioredoxin reductase	Pamp et al. [35]
		icaABCD	Biofilm formation	
		trfA	β-lactam resistance	Wang et al. [34]

Adapted from Hillion and Antelmann (2015)

Another important regulator found to be upregulated is SarA (staphylococcal accessory regulator), a global redox-sensing regulator that positively regulates many virulence factors and controls oxidative stress-related genes. Additionally, Spx (encoded by *spxA*), which functions as the global regulator of genes that maintain the thiol-redox balance, and is required for transcription of *trxB* encoding thioredoxin reductase, was also found to be upregulated in the sensitized cells (Table 1) [20,33–35]. Staphylococci use several enzymes for the detoxification of reactive oxygen, including alkyl hydroperoxide reductase (Ahp) and superoxide dismutases (SODs) (induced by the sensors SarA and Spx) and most prominently catalase (KatA), which is transcriptionally regulated by PerR and Fur in response to peroxide; all of these enzymes were upregulated in the sensitized cells [20,35,43,44]. Combined, the findings from the transcriptional analysis indicated that activation of the oxidative-stress protection system may, in fact, confer the cell with enhanced tolerance to oxidizing agents. This hypothesis was validated using H_2_O_2_ killing assays where the sensitized cells were shown to exhibit higher tolerance to the oxidizing agent. More importantly, the farnesol-sensitized cells also demonstrated an increase in survival within phagocytic cells.

In addition to these enzymes, *S. aureus* rely on the thioredoxin system comprised of cysteine-containing small proteins, thioredoxin (*trxA*) and thioredoxin reductase (*trxB*) to carry out the thiol-disulfide redox cycling reactions [20]. Therefore, cysteine biosynthesis is critical and oxidative-stress increases cysteine biosynthesis and uptake, thus, it was not surprising to find *cysK*, the gene encoding cysteine synthase to be upregulated in the farnesol-sensitized cells (Table 1). Once ROS mediated cell damage occurs, *S. aureus* cells express several enzymes including thioredoxin/thioredoxin reductases (Trx) and methionine sulfoxide reductases (Msr) to repair protein damage, both of which were upregulated in response to farnesol.

Another group of genes that were notably differentially regulated by farnesol are those involved in metal homeostasis (Table 1). Metal ions such as iron (Fe) and copper (Cu) act as cofactors for enzymes and are essential for electron transfer; however, this ability also facilitates the generation of ROS through Fenton chemistry [45]. To maintain intracellular metal ion homeostasis, bacteria utilize active transporters, efflux systems, and metallochaperones [45]. The iron-regulated surface determinants (Isd) involved in iron acquisition and transport (*isdA, isdB, isdCDEF, isdG, isdH*, and *isdI)* are regulated by Fur, which mainly functions as a repressor of iron-responsive genes [46,47]. After the acquisition, iron is utilized or bound by ferritin, the major iron storage protein and scavenger of intracellular iron, or Dps (DNA-binding protein from starved cells), which also functions as iron chelator/storage protein [20,37]. In our analyses, the repressor Fur was found to be upregulated, whereas proteins involved in iron binding and transport were downregulated (Table 1). Collectively, these findings indicate inactivation of the iron uptake system due to an increase in intracellular free iron, or attenuation to prevent further oxidative damage. Similar to iron, copper (Cu) can also be toxic through the generation of ROS; in *S. aureus*, copper homeostasis is primarily maintained by the P1-type ATPase CopA Cu exporter, which we also found to be upregulated [20,47] (Table 1).

In cells, methionine is highly susceptible to oxidation, which can lead to inhibition of enzymatic functions and therefore, *S. aureus* rely on methionine sulfoxide reductases (*msrA1, msrA2, msrA3, msrB* genes) for repair [48]. The oxidized form, methionine sulfoxide is reduced by the enzyme MsrB, and the reduction is dependent on the thioredoxin thiol-disulfide redox system [48]. Therefore, the increased expression of *msrA1* and *msrB* seen in the farnesol-sensitized cells indicate induction of protein damage by farnesol [20]. Similarly, iron-sulfur (Fe-S) clusters involved in diverse cellular processes are also susceptible to oxidative inactivation and therefore, bacteria have evolved Fe-S cluster repair mechanisms. In *S. aureus*, transcription of *scdA*, the Fe-S cluster repair protein, can be induced by exposure to H_2_O_2_ [20,38] and this protein was found to be upregulated indicating farnesol-induced damage to Fe-S clusters.

Oxidative damage to DNA can produce strand breakage or base alterations and therefore, DNA repair mechanisms have evolved to maintain genetic integrity. The *lexA* gene encodes a repressor protein regulating the SOS response genes, which include DNA repair genes *via* initiation of RecF and SbcCD pathways, followed by RecA binding to DNA [20,31]. During an SOS response, the sensor protein RecA becomes activated and stimulates the autocatalytic cleavage of the SOS transcriptional repressor LexA leading to the de-repression of SOS genes [20,31]. The upregulation of RecF and RecA in addition to Dps, which is also involved in protecting DNA [49], indicates farnesol-induced DNA damage and activation of the SOS response.

In addition to detoxifying enzymes, carotenoid pigments are considered important antioxidants and therefore, it is expected that STXN would be impacted during the process of detoxification. However, where farnesol exposure resulted in dramatic and complete depigmentation, no major change in pigmentation was seen when cells were oxidatively stressed by H_2_O_2_ indicating that loss of pigment is primarily mediated by farnesol. Interestingly, RNA sequencing indicated no alterations in the regulation of the *crtM* and *crtN* genes, excluding transcriptional modulations in the STXN biosynthesis pathway as a mechanism for STXN inhibition (Table 1).

**Figure 9.**
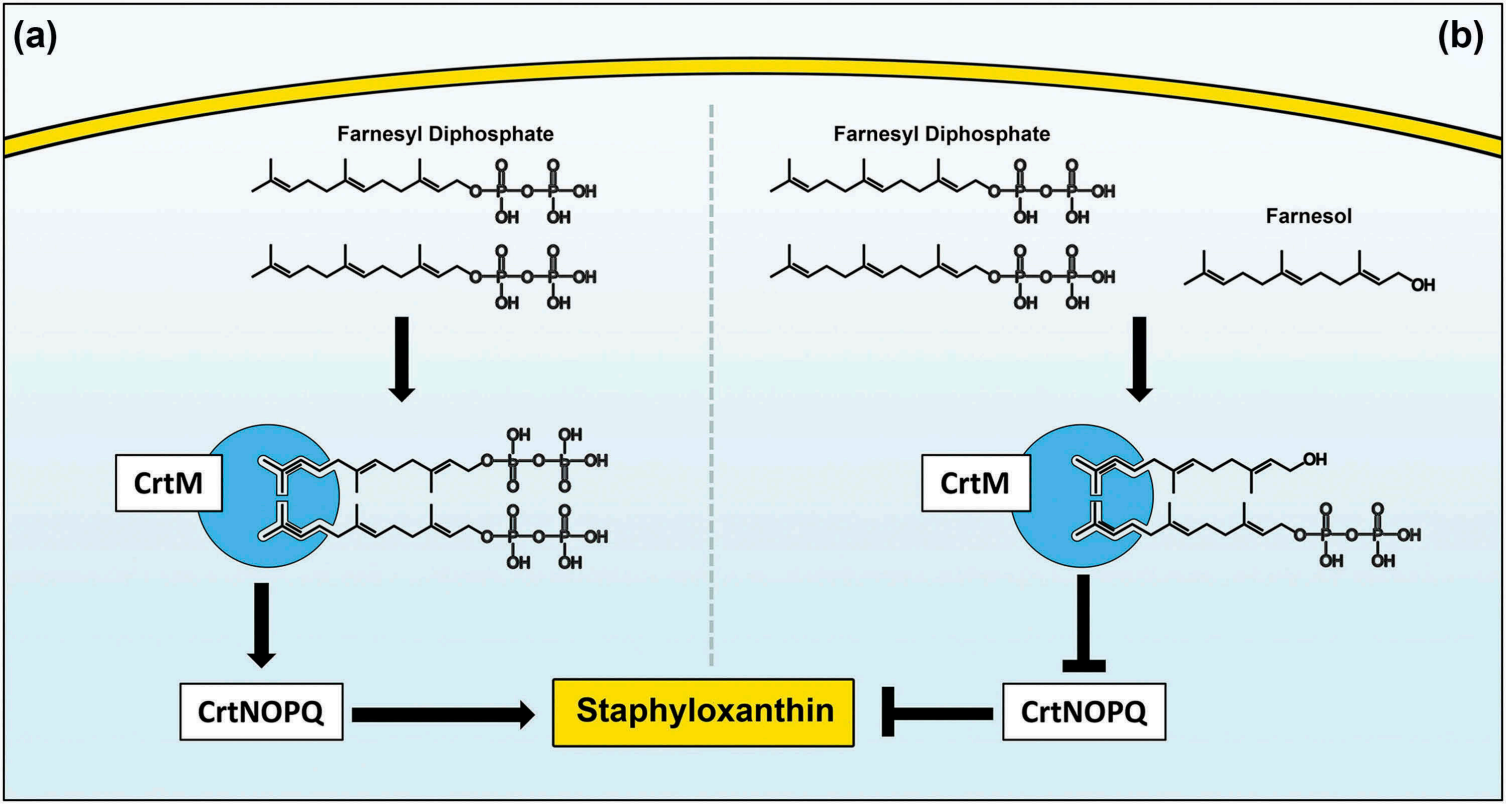
Proposed model for the inhibition of staphyloxanthin production in *S. aureus* by farnesol. (a) Staphyloxanthin biosynthesis begins with the CrtM enzyme binding to its substrate farnesyl diphosphate (FPP), which involves the head-to-head condensation of two molecules of FPP to form dehydrosqualene, eventually yielding staphyloxanthin. (b) However, due to close structural homology to FPP, farnesol competes with FPP for CrtM binding by occupying the catalytic site on the enzyme. Since farnesol lacks the required diphosphate groups, the CrtM enzyme is unable to complete the condensation reaction to synthesize dehydrosqualene, thus halting staphyloxanthin synthesis.

In *S. aureus*, STXN biosynthesis pathway begins with CrtM binding to its substrate farnesyl diphosphate (FPP), which involves the head-to-head condensation of two molecules of FPP, eventually yielding STXN [23]. Interestingly, FPP bears remarkable structural homology to farnesol, suggesting that farnesol can also occupy the catalytic site on the enzyme (Figure 9). However, since farnesol lacks the required diphosphate groups, the CrtM enzyme is unable to complete the condensation reaction, thus halting STXN synthesis. To explore competitive binding as a potential mechanism for STXN inhibition, using computer simulation software and the CrtM-FPP model, we generated a computational theoretical CrtM-farnesol binding model, which indeed indicated that the majority of the CrtM binding interface is conserved between the two models (Figure 7). Although theoretical, this modeling strongly supports the hypothesis that the inhibition of STXN is at least partly due to farnesol competitive binding to the CrtM enzyme, which is corroborated by the observed farnesol dose-dependent loss of STXN (Figure 1(a)).

In summary, the findings from this study demonstrate that a *C. albicans*-secreted quorum-sensing molecule exerts oxidative stress on *S. aureus* eliciting a stress response *via* activation of the thiol-based redox system under control of global regulators of virulence. Intriguingly, these findings combined with those from our previous studies indicate a conserved mechanism for farnesol’s effect on prokaryotic and eukaryotic cells, both fungal and mammalian, involving disruption of the intracellular thiol-based redox-balance. However, in eukaryotic cells, we identified the mechanism to involve depletion of the thiol glutathione (which *S. aureus* lacks), a process mediated by conjugation of farnesol with reduced glutathione and efflux of the farnesol-oxidized glutathione conjugate complex by the ABC transporters *CDR1* and *MDR1*, in *C. albicans* and mammalian cells, respectively [50,51].

Of most significance are the findings exploring the impact of physiologically secreted farnesol on *S. aureus* behavior during co-growth. Combined, the findings demonstrated that similar to exogenous farnesol supplementation, *S. aureus* growth with *C. albicans* resulted in a significant loss in cell pigmentation but not when grown with a farnesol-deficient *C. albicans* strain (Figure 8). Identification of farnesol as the key effector impacting pigmentation was supported by HPLC analysis demonstrating the presence of farnesol in the spent biofilm culture media of the farnesol-producing but not the farnesol-deficient *C. albicans* strain. Therefore, based on the combined findings from this study, it is tempting to speculate that farnesol-induced modulations of key regulatory networks may impact *S. aureus* fitness and virulence. Whether *C. albicans* influences *S. aureus* cell behavior under *in vivo* conditions, however, warrants an understanding of the transcriptome of *S. aureus* during the course of a co-infection. To that end, studies using mouse models of biofilm-associated polymicrobial infections are currently underway in our laboratory.

## Materials and methods

### Reagents

Trans-trans farnesol was purchased from Sigma Aldrich (St. Louis, MO) as a 3 M stock solution and diluted to a 30 mM solution in 100% methanol. Methanol was included in all control experiments. Hydrogen peroxide, catalase, and lysostaphin were purchased from Sigma-Aldrich (USA) and the CellROX^TM^ green reagent for ROS detection was purchased from Invitrogen (Thermo Fisher Scientific, USA).

### Strains and growth conditions

The *S. aureus* standard methicillin-resistant *S. aureus* (MRSA) wild-type strain USA300, the *S. aureus* NE1444 *crtM* deletion strain (Nebraska Transposon Mutant Library) and the *C. albicans* SC5314 reference strain [52] and the ATCC10231 (wild-type farnesol-deficient) strain [11,53] were used in these studies. *C. albicans* strains were maintained on YPD agar and for experiments, grown for 16 hrs in YPD broth at 30°C. *S. aureus* was maintained on TSA agar and grown in TSB broth for 16 hrs at 37°C. For biofilm experiments, cells were re-suspended in RPMI 1640 with L-glutamine and HEPES (Invitrogen, Grand Island, NY).

### Murine macrophages

Cells of the RAW 264.7 macrophage cell line from the American Type Culture Collection were maintained in DMEM High Glucose (with L-Glutamine and sodium pyruvate) medium supplemented with 10% BenchMark^TM^ Fetal Bovine Serum (FBS; Gemini Bio-Products, USA). Macrophages were cultured in T-75 culture flasks at 37°C in the presence of 5% CO_2_ and passaged for no more than 6 weeks at which time new cells were revived from liquid nitrogen frozen stocks.

### Farnesol dose-dependent effect on cell pigmentation

*S. aureus* cells were grown overnight as described above in TSB supplemented with farnesol to final concentrations of 30, 50, 100 and 200 µM. Control cells were grown in the absence of farnesol in the same conditions. Cells were harvested, washed with PBS and visually evaluated for pigment accumulation.

### Generation of a depigmented *S. aureus* phenotype upon gradual repeated exposure of cells to farnesol (sensitization)

*S. aureus* cells were grown overnight as described above in TSB supplemented with farnesol to a final concentration of 50 µM (FP1) or with no farnesol (P1) (first Passage). The passaging of cells into their respective cultures (with and without farnesol) was repeated two more times (second and third Passage) to generate farnesol-sensitized cells following the third passage over a 3-day period. The fully “sensitized” cells from the final passage (FP3) were washed and used in subsequent experiments. Control cells not exposed to farnesol were simultaneously passaged during the sensitization process (P1-P3). Cells from each passage were harvested, washed with PBS, evaluated and used in subsequent experiments.

### Restoration of color in the depigmented sensitized cells upon gradual passaging in farnesol-free media (desensitization)

A desensitization process was performed on the fully sensitized *S. aureus* cells following the same scheme as for sensitization; sensitized cells from “3^rd^ Passage” (FP3) were grown in fresh TSB (with no farnesol) and transferred daily to fresh media in the absence of farnesol until the fifth Passage (FP5) at which time the yellow color was fully restored and cells were considered desensitized. Control cells not exposed to farnesol from the sensitization process were simultaneously passaged during desensitization (P1-P5). Cells from each passage were harvested, washed with PBS and evaluated.

### STXN detection in farnesol-sensitized and control *S. aureus* cells by HPLC analysis

Sensitized and desensitized cells and their respective controls were harvested and washed in PBS. Extraction, purification, and HPLC analysis were performed as previously described [23]. Briefly, cell pellets were resuspended in 20 mL of 100% ethanol and suspensions were incubated at 40°C on an orbital shaker for 40 min. Cell suspensions were centrifuged and supernatant collected and filter sterilized (0.22 µm pore). 5 mL of the generated crude ethanolic extracts containing the pigments were subsequently extracted with ethyl acetate/1.7 M aqueous NaCl (1:1, v/v). The colored ethyl acetate extracts were dried with anhydrous Na_2_SO4, and the solvent was evaporated under a nitrogen stream. The residue was dissolved in 300 L of acetonitrile and 50 L of the solution were injected unto UPLC. RP-HPLC analysis was carried out on a UPLC sing ACQUITY UPLC H-Class System with Fluorescence Detection (Waters Corp., Milford, MA, USA) using XBridge UPLC C-18 column, 4.6 × 100 mm, 3.5 µm (Waters Corp., Milford, MA, USA). Compounds were separated with an Acetonitrile/water gradient (0 min, 55% Acetonitrile; 0–10 min, linear gradient to 95% Acetonitrile; 10–30 min, 95% Acetonitrile) at a flow rate of 1 ml/min. Analyses were performed on three sets of biological samples processed on three separate occasions.

### Farnesol-induced accumulation of intracellular ROS and loss of cell pigment

*S. aureus* cells were grown for 3 hrs or 18 hrs at 37°C with shaking in TSB supplemented with farnesol at a final concentration of 50 µM or H_2_O_2_ (final concentration 150 mM). Cells grown in the absence of farnesol or H_2_O_2_ were used as control. Following incubation, cells were harvested, washed twice in PBS and standardized to 1 × 10^8^ cells/mL in RPMI. CellROX green reagent was added to a final concentration of 5 µM and cells were incubated for 30 min at 37°C. Cells were then washed with PBS and ROS accumulation was quantified using a fluorescent plate reader (Cytation5, BioTek) at excitation/emission: 485/535 nm.

### Quantitative evaluation of cell pigmentation

Cell pigment accumulation was evaluated as described previously [26]. Briefly, 8 mL of the cultures were washed in PBS and the pellets were weighed for normalization. Cells were resuspended in 2 mL 100% methanol and incubated for 20 min in a water bath at 45–50°C. Following incubation, cells were centrifuged at 10,000 RPM for 15 min and the absorbance of the supernatant was measured at 450nm. Pigment concentration was normalized by the biomass in grams form each culture.

### Assessment of growth rate, adhesion and biofilm formation of the farnesol-sensitized *S. aureus*

To assess if farnesol exerts an adverse effect on the cells, the growth rate was evaluated during the sensitization of cells. *S. aureus* cells were grown for 24 hrs at 37°C, as described above in TSB supplemented with farnesol to final concentration of 50 µM (FP1, FP2 and FP3) or with no farnesol (P1, P2 and P3) in a final cell density of 6 × 10^6^ cells/mL, in order to generate all three steps of the sensitized phenotype in parallel. During growth, aliquots of 10 µL were diluted in 900 µL of PBS and the optical density was measured at 600 nm, every 2 h. For adhesion and biofilm formation, cell suspensions (100 µL) in RPMI (1 × 10^6^ cells/mL) were added to the wells of a 96-well plate. Plates were incubated at 37°C for 90 min to evaluated adhesion and for 24 hrs for biofilm formation. Following incubation, wells were washed with PBS, 100 µl of PBS was added to the wells followed by 20 µL of MTS reagent and plates were incubated at 37°C until color fully developed. Colorimetric change at 490 nm (*A*_490_) was measured with a microtiter plate reader. For confirmation, some wells were sonicated to release adherent cells and suspensions were diluted in PBS and plated on bacterial media for CFUs enumeration.

### Transmission electron microscopy (TEM)

Since STXN accumulates in the cell wall, cells were evaluated by TEM to exclude pigment leakage as a cause for loss of pigment. Briefly, cells were fixed, embedded in agarose and blocks were post-fixed with 1% osmium tetroxide/1.5% potassium ferrocyanide then stained with uranyl acetate. Specimens were serially dehydrated in ethanol, and embedded in Spurr resin (Electron Microscopy Sciences, PA). Ultrathin sections (~70nm) mounted on copper grids were examined in a Tecnai T12 TEM (Thermo Fisher Scientific, Hillsboro, OR). Digital images were taken using a CCD camera (Advanced Microscopy Techniques, Corp, Woburn, MA) and AMT600 software.

### Assessment of cell membrane integrity

Cells grown for 16 hrs in the absence or presence of farnesol were harvested, washed with PBS twice and normalized to 1 × 10^8^ cells/mL in PBS. Cells were stained with 5 µM of propidium iodide (PI) for 20 min at 37°C. Subsequently, cells were washed twice in PBS and 100 µL of each sample was transferred to a 96-well plate for fluorescence quantification at excitation/emission of 493/636nm (Cytation5, BioTek). Cells exposed for 20 min to 70% ethanol were used as positive controls.

### RNA-sequencing and gene expression analysis

Total RNA was extracted from farnesol-sensitized (FP3) and control cells and rRNA was depleted with the RiboZero Gram-positive depletion kit (Illumina). All RNA-seq libraries (strand-specific, paired end) were prepared with the TruSeq RNA sample prep kit (Illumina). One hundred and fifty nucleotides of the sequence were determined from both ends of each cDNA fragment using the HiSeq platform (Illumina). Sequencing reads were aligned to the reference *S. aureus* genome (*Staphylococcus aureus* USA300 TCH1516 uid58925) using HISAT [54] and alignment files were used to generate read counts for each gene; statistical analysis of differential gene expression was performed using the DEseq package from Bioconductor [55]. A gene was considered differentially expressed if the absolute log-fold change ≥1 and the P-value for differential expression was less than 0.05. Two biological samples were subjected for RNA-seq analysis.

### Susceptibility of farnesol-sensitized *S. aureus* to hydrogen peroxide

*S. aureus* sensitized cells from third passage were grown without (P3) or with 50 µM farnesol (FP3) and suspensions were adjusted to a final cell density of 1 × 10^8^ cells/mL in PBS as previously described. Cell suspensions (10 µL) were added to the wells of 96-well plates with 90 µL of an H_2_O_2_ solution (100 mM) or PBS (control) and plates were incubated for 1 hr at 37°C. Reactions were stopped by the addition of 1 µL of catalase (200 KU/mL) and following incubation for 20 min at room temperature, suspensions were serially diluted in PBS and spotted (10 µL) on TSA agar plates and incubated for 24 hrs at 37°C for killing/growth evaluation.

### Phagocytosis assay

RAW macrophages (5 × 10^5^ cells/mL) were seeded in the wells of 12-well tissue culture plates and incubated overnight at 37°C with 5% CO_2_. Cultures of sensitized (FP3) and control (P3) *S. aureus* cells grown as previously described were washed with sterile PBS and diluted in DMEM supplemented with 10% FBS. Macrophages were infected at a MOI of 10:1 and plates were incubated for 30 min at 37°C with 5% CO_2_ to allow for phagocytosis. Following ingestion of bacterial cells, monolayers were gently rinsed with PBS to remove non-phagocytosed cells and fresh media containing lysostaphin (10 µg/mL) was added and plates were incubated for 1 hr to eliminate non-phagocytosed cells. Following lysostaphin treatment, macrophages were rinsed with PBS and incubated in fresh media for an additional 2–3 hrs upon which infected macrophages were harvested and lysed by the treatment with 0.1% (v/v) Triton X-100 in PBS to release the phagocytosed *S. aureus* cells. Macrophage lysates were serially diluted, plated on tryptic soy agar plates and incubated at 37°C for 24 hrs and survival of released intracellular *S. aureus* cells was assessed based on CFUs enumeration.

### Modeling farnesol binding to CtrM

Using AMBER ff14SB with a TIP3P water model [56], the energy values for the CrtM-FPS (farnesyl thiopyrophosphate) and CrtM-FOH (farnesol) complexes were compared with their free equivalents. Each structure was parametrized with hydrogens added using ANTEChamber to assign partial charges for the farnesyl pyrophosphate and farnesol ligands, and encased in a 6Å solvent shell (with the magnesium ions preserved in the CrtM-FPS structure) [57]. Structures were then minimized with the steepest descent gradient of six 0.2Å steps.

### Effect of mixed biofilm growth on *S. aureus* cell pigmentation and ROS accumulation

Biofilms were formed by inoculating 40 mL of RPMI in canted-neck cell culture flasks (150 cm^2^) with *S. aureus* at a final cell density of 1 × 10^6^ cells/mL with and without *C. albicans* at 1 × 10^6^ cells/mL final cell density. Biofilms were allowed to form for 48 hrs at 37°C then harvested by scraping. Cells were sonicated in a warm water bath for 30 min to dissociate *S. aureus* cells adhering to *C. albicans* hyphae; then cell suspensions were vigorously vortexed for 30 sec and immediately filtered through a 5 µm pore filter to remove *C. albicans* and isolate *S. aureus* cells. Mono-species *S. aureus* biofilms were similarly grown and filtered. Recovered *S. aureus* cells were washed in PBS and pigment was extracted and quantified as described above. In parallel, ROS accumulation was quantified as described above using CellROX green reagent. Cell suspensions were plated (post-filtration) on species-specific chromogenic agar (CHROMagar) to confirm the removal of fungal cells and isolation of bacterial cells.

### Comparative measurement of farnesol secreted by the *C. albicans* strains during biofilm growth by HPLC analysis

Biofilms of the farnesol-producing and deficient *C. albicans* strains were grown statically in RPMI for 48 hrs at 37°C. Recovered cell-free culture spent media was filter-sterilized and subjected to HPLC analysis using ACQUITY UPLC H-Class System with Fluorescence Detection (Milford, MA) and XBridge UPLC C-18 column, 4.6 × 100 mm, 3.5 μm. UV detection was done at 210 nm and total chromatographic run time was 35 min. Injection volume was 20.0 µL and farnesol retention time 12.29 min.

### Effect of co-growth with a farnesol-deficient *C. albicans* strain on *S. aureus* pigmentation

*S. aureus* cultures were grown planktonically in TSB as previously described with the addition of *C. albicans* cells to a final cell density of 1 × 10^6^ cells/ml for both organisms. *S. aureus* was similarly grown alone in culture as a control. Following incubation, cultures were washed in PBS, vigorously vortexed for 30 sec and immediately filtered through a 5 µm pore filter and *S. aureus* cells were recovered (filtrate was cultured to confirm the absence of *C. albicans*). The pigment was extracted as described above from *S. aureus* cells harvested from single and mixed cultures.

### Data analysis

All experiments were performed on at least three separate occasions and in triplicate where applicable, and averages of data sets are presented. Statistical analysis was performed using GraphPad Prism 5.0 software. The Kruskal–Wallis one-way analysis of variance test was used to compare differences between multiple groups, and Dunnet’s multiple-comparison test to determine whether differences between two samples are statistically significant. Student’s unpaired *t* test was used to compare differences between two samples. *P* values of <0.05 were considered to be significant.
