# Supplementary material for: Candida albicans quorum-sensing molecule farnesol modulates staphyloxanthin production and activates the thiol-based oxidative-stress response in Staphylococcus aureus
Source: Virulence. 2019 Jul 6;10(1):625–42. doi: 10.1080/21505594.2019.1635418 (PMC6629188; doi:10.1080/21505594.2019.1635418)
Supplement: Supplemental Material [file kvir-10-01-1635418-s001.docx]

**Supplementary Table 1.** Conserved contacts between CrtM and the Farnesyl Backbone

| **Atom1** | **Atom2** | **VdW overlap** | **Distance (A)** |
| --- | --- | --- | --- |
| FPS 301.A H6 | ASP 48.A HB2 | 0.368 | 1.632 |
| FPS 301.A C7 | CYS 44.A HB3 | 0.297 | 2.403 |
| FPS 301.A C13 | TYR 41.A HD2 | 0.236 | 2.464 |
| FPS 301.A H6 | ASP 48.A CB | 0.156 | 2.544 |
| FPS 301.A C6 | ASP 48.A HB2 | 0.128 | 2.572 |
| FPS 301.A C14 | LEU 141.A HD11 | 0.114 | 2.586 |
| FPS 301.A C10 | CYS 44.A CB | 0.112 | 3.288 |
| FPS 301.A C8 | CYS 44.A HB3 | 0.058 | 2.642 |
| FPS 301.A H2 | ASP 48.A CG | 0.042 | 2.658 |
| FPS 301.A H12 | VAL 137.A HG13 | 0.031 | 1.969 |
| FPS 301.A C8 | CYS 44.A CB | 0.007 | 3.393 |
| FPS 301.A C12 | TYR 41.A HD2 | 0.004 | 2.696 |
| FPS 301.A C15 | TYR 41.A HD2 | -0.005 | 2.705 |
| FPS 301.A C12 | VAL 137.A HG13 | -0.014 | 2.714 |
| FPS 301.A H141 | LEU 141.A HD11 | -0.03 | 2.03 |
| FPS 301.A C7 | CYS 44.A CB | -0.033 | 3.433 |
| FPS 301.A C6 | ASP 48.A CB | -0.074 | 3.474 |
| FPS 301.A C13 | TYR 41.A CD2 | -0.076 | 3.476 |
| FPS 301.A C15 | TYR 41.A CD2 | -0.082 | 3.482 |
| FPS 301.A H12 | VAL 137.A CG1 | -0.1 | 2.8 |
| FPS 301.A C10 | CYS 44.A HB2 | -0.108 | 2.808 |
| FPS 301.A C12 | TYR 41.A CD2 | -0.135 | 3.535 |
| FPS 301.A H103 | CYS 44.A HB2 | -0.155 | 2.155 |
| FPS 301.A C10 | CYS 44.A HB3 | -0.175 | 2.875 |
| FPS 301.A H152 | MET 15.A HE2 | -0.177 | 2.177 |
| FPS 301.A H2 | ASP 48.A OD2 | -0.182 | 2.662 |
| FPS 301.A C15 | TYR 41.A CE2 | -0.204 | 3.604 |
| FPS 301.A C11 | TYR 41.A CD2 | -0.211 | 3.611 |
| FPS 301.A H152 | MET 15.A CE | -0.219 | 2.919 |
| FPS 301.A H1 | ARG 45.A HH22 | -0.226 | 2.226 |
| FPS 301.A C11 | TYR 41.A CE2 | -0.227 | 3.627 |
| FPS 301.A H103 | CYS 44.A CB | -0.242 | 2.942 |
| FPS 301.A C14 | LEU 141.A CD1 | -0.254 | 3.654 |
| FPS 301.A H52 | ASP 48.A OD1 | -0.268 | 2.748 |
| FPS 301.A H151 | TYR 41.A HE2 | -0.28 | 2.28 |
| FPS 301.A H53 | VAL 133.A O | -0.281 | 2.761 |
| FPS 301.A H143 | LEU 141.A HD11 | -0.285 | 2.285 |
| FPS 301.A C12 | VAL 137.A CG1 | -0.288 | 3.688 |
| FPS 301.A C15 | TYR 41.A HE2 | -0.29 | 2.99 |
| FPS 301.A H7 | CYS 44.A HB3 | -0.305 | 2.305 |
| FPS 301.A C2 | ASP 48.A CG | -0.313 | 3.713 |
| FPS 301.A H102 | CYS 44.A CB | -0.314 | 3.014 |
| FPS 301.A H6 | CYS 44.A O | -0.318 | 2.798 |
| FPS 301.A C9 | ARG 45.A NH2 | -0.344 | 3.669 |
| FPS 301.A H152 | TYR 41.A CD2 | -0.369 | 3.069 |
| FPS 301.A H141 | LEU 141.A CD1 | -0.396 | 3.096 |
| FPS 301.A H152 | TYR 41.A HD2 | -0.398 | 2.398 |

**Supplementary Table 2.** Conserved hydrogen bonds between FPP-CrtM and Farnesol-CrtM

| **Donor Heavy Atom** | **Acceptor Heavy Atom** | **D.A. Hydrogen** | **D.A Distance** | **D-H…A Distance** |
| --- | --- | --- | --- | --- |
| ASN 7.A ND2 | HOH 416.A O | ASN 7.A HD22 | 3.26 | 2.35 |
| LYS 13.A NZ | HOH 480.A O | LYS 13.A HZ1 | 2.109 | 1.244 |
| LYS 16.A NZ | HOH 535.A O | LYS 16.A HZ2 | 2.169 | 1.236 |
| TYR 24.A N | HOH 493.A O | TYR 24.A H | 3.037 | 2.042 |
| GLU 31.A N | HOH 567.A O | GLU 31.A H | 3.182 | 2.237 |
| ASP 32.A N | HOH 547.A O | ASP 32.A H | 2.958 | 1.955 |
| LYS 46.A NZ | HOH 428.A O | LYS 46.A HZ3 | 2.806 | 1.825 |
| HIS 78.A N | HOH 418.A O | HIS 78.A H | 3.007 | 2.002 |
| GLN 81.A NE2 | HOH 472.A O | GLN 81.A HE22 | 2.885 | 1.913 |
| ARG 85.A N | HOH 413.A O | ARG 85.A H | 3.366 | 2.369 |
| ARG 85.A NE | HOH 413.A O | ARG 85.A HE | 3.129 | 2.131 |
| ARG 85.A NH1 | HOH 414.A O | ARG 85.A HH11 | 2.75 | 1.823 |
| GLN 102.A NE2 | HOH 470.A O | GLN 102.A HE22 | 3.303 | 2.314 |
| TYR 129.A OH | HOH 440.A O | TYR 129.A HH | 2.792 | 1.846 |
| GLY 174.A N | HOH 439.A O | GLY 174.A H | 3.194 | 2.219 |
| TYR 183.A N | HOH 420.A O | TYR 183.A H | 2.404 | 1.421 |
| ALA 197.A N | HOH 474.A O | ALA 197.A H | 3.414 | 2.416 |
| TYR 248.A OH | FPS 302.A O1B | TYR 248.A HH | 2.996 | 2.146 |
| ARG 265.A N | HOH 551.A O | ARG 265.A H | 3.306 | 2.379 |
| ARG 265.A NH2 | HOH 406.A O | ARG 265.A HH21 | 2.786 | 1.865 |
| GLU 269.A N | HOH 484.A O | GLU 269.A H | 2.999 | 2.051 |
| LYS 270.A NZ | HOH 489.A O | LYS 270.A HZ1 | 3.328 | 2.433 |
| LYS 273.A NZ | HOH 465.A O | LYS 273.A HZ3 | 2.764 | 1.796 |
| HOH 423.A O | TYR 131.A OH | HOH 423.A H1 | 3.517 | 2.56 |
| HOH 425.A O | TYR 208.A OH | HOH 425.A H1 | 2.597 | 1.671 |
| HOH 449.A O | SER 69.A OG | HOH 449.A H1 | 2.852 | 1.895 |
| HOH 488.A O | SER 282.A OG | HOH 488.A H1 | 3.189 | 2.231 |
| HOH 492.A O | LEU 170.A O | HOH 492.A H2 | 2.815 | 2.095 |
| HOH 493.A O | SER 19.A O | HOH 493.A H2 | 3.461 | 2.506 |
| HOH 508.A O | HIS 79.A ND1 | HOH 508.A H1 | 2.632 | 1.675 |
| HOH 513.A O | GLU 242.A OE1 | HOH 513.A H2 | 3.435 | 2.668 |
| HOH 523.A O | TYR 77.A OH | HOH 523.A H2 | 2.688 | 1.813 |
| HOH 527.A O | HIS 263.A NE2 | HOH 527.A H1 | 2.604 | 1.647 |
| HOH 528.A O | HIS 18.A NE2 | HOH 528.A H1 | 2.68 | 1.724 |
| HOH 530.A O | THR 2.A OG1 | HOH 530.A H1 | 2.738 | 1.781 |
| HOH 554.A O | SER 146.A OG | HOH 554.A H1 | 3.041 | 2.084 |

**Supplementary Table 3.** FPP-CrtM hydrogen bonds lost in the Farnesol-CrtM model

| **Donor Heavy Atom** | **Acceptor Heavy Atom** | **D.A. Hydrogen** | **D.A. Distance** | **D-H…A Distance** |
| --- | --- | --- | --- | --- |
| MET 3.A N | HOH 410.A O | MET 3.A H | 3.153 | 2.147 |
| SER 21.A N | FPS 302.A O2B | SER 21.A H | 3.185 | 2.215 |
| ARG 45.A NH1 | FPS 301.A O2B | ARG 45.A HH12 | 3.428 | 2.662 |
| ARG 45.A NH1 | FPS 301.A O3B | ARG 45.A HH12 | 2.585 | 1.671 |
| ARG 45.A NH2 | FPS 301.A O2B | ARG 45.A HH22 | 3.175 | 2.322 |
| ARG 45.A NH2 | FPS 302.A O1A | ARG 45.A HH22 | 3.052 | 2.282 |
| ARG 171.A NH1 | FPS 302.A O1B | ARG 171.A HH12 | 2.685 | 1.759 |
| ARG 171.A NH1 | FPS 302.A O3B | ARG 171.A HH12 | 3.412 | 2.573 |
| ARG 171.A NH2 | FPS 302.A O3B | ARG 171.A HH22 | 3.129 | 2.189 |
| ARG 271.A N | HOH 499.A O | ARG 271.A H | 2.836 | 1.849 |
| HOH 559.A O | MET 6.A O | HOH 559.A H1 | 3.101 | 2.345 |
| HOH 562.A O | TYR 75.A OH | HOH 562.A H1 | 3.426 | 2.477 |

**Supplementary Table 4.** Novel hydrogen bonds in the Farnesol-CrtM model

| **Donor Heavy Atom** | **Acceptor Heavy Atom** | **D.A. Hydrogen** | **D.A Distance** | **D-H…A Distance** |
| --- | --- | --- | --- | --- |
| ARG 45.A NH2 | HOH 2.B O | ARG 45.A HH22 | 3.052 | 2.282 |
| ASP 147.A N | HOH 554.A O | ASP 147.A H | 3.057 | 2.058 |
| ASN 168.A ND2 | HOH 4.B O | ASN 168.A HD22 | 3.388 | 2.397 |
| ARG 171.A NH1 | HOH 11.B O | ARG 171.A HH11 | 2.685 | 1.759 |
| HOH 1.B O | TYR 41.A OH | HOH 1.B H2 | 2.585 | 1.655 |
| HOH 11.B O | SER 21.A OG | HOH 11.B H1 | 2.405 | 1.46 |
